# 

**Published:** 1880-01

**Authors:** 


Will be supplied with Hall's Journal of Health by "The American News Company" and the "New York News Co." at reduced rates, commencing with January, 1880.

Post Masters, Publishers, Booksellers, and others, acting as Agents, can retain 50 cents out of each yearly subscription they send to us. No larger commission will be allowed in any case.

E. H. GIBBS, A.M., M.D., Editor,

No. 141 EIGHTH STREET (near Broadway) NEW YORK.

Terms, $1.50 a Year, or $1.00 for Eight Months. Postage paid.

Entered as Second-Class matter in the Post-office in New York.

### BOOK NOTICES.

*Practical Boat Sailing.* By Douglas Frazar.

This little volume contains all the instructions necessary to enable one to manage a yacht with safety and pleasure to himself. The text is illustrated by diagrams representing the most approved forms of rigging, and also plans of various nautical maneuvers. A vocabulary of sea terms is added. Price, $1.

LEE & SHEPARD, Publishers, Boston, Mass.

---

*A Tight Squeeze.* By Staats.

An entertaining book, relating the adventures of a gentleman who, on a wager of $10,000, undertook to walk from New York to New Orleans in three weeks, without money or the assistance of friends. Price, $1.

LEE & SHEPARD, Publishers, Boston, Mass.

---

*Daisy Travers*, by Adelaide F. Samuels, and
*That Queer Girl*, by Virginia F. Townsend,

Are two juvenile books of the "Maidenhood Series." They are both full of incidents interesting to the young mind. Price, handsomely bound, $1.50 each.

LEE & SHEPARD, Publishers, Boston, Mass.

---

AFTER THE numerous discussions of Mr. Edison's electric light, it will be interesting to see exactly what claims for it Mr. Edison himself is willing to indorse. A paper is announced to appear in the midwinter *Scribner*, by Mr. Edison's mathematician and assistant, Mr. Francis R. Upton, which, besides the writer's intimate connection with the inventor himself, has the further voucher of a letter from Mr. Edison, certifying that it is "the first correct and authoritative account." It is said that the paper will contain much that has not been and will not be elsewhere published.

AMONG the useful papers promised for the February *St. Nicholas* will be one on the Audiphone, that recent and admirable invention by which persons, so deaf that they have never heard a sound in their lives, can be made to hear music, the human voice, and all the beautiful sounds of nature. This paper will doubtless be of interest to old and young.

